# A biomechanical-based approach to scale blast-induced molecular changes in the brain

**DOI:** 10.1038/s41598-022-17967-6

**Published:** 2022-08-26

**Authors:** Jose E. Rubio, Dhananjay Radhakrishnan Subramaniam, Ginu Unnikrishnan, Venkata Siva Sai Sujith Sajja, Stephen Van Albert, Franco Rossetti, Andrew Frock, Giang Nguyen, Aravind Sundaramurthy, Joseph B. Long, Jaques Reifman

**Affiliations:** 1 Department of Defense Biotechnology High Performance Computing Software Applications Institute, Telemedicine and Advanced Technology Research Center, United States Army Medical Research and Development Command, ATTN: FCMR-TT, 504 Scott Street, Fort Detrick, MD 21702-5012 USA; 2 The Henry M. Jackson Foundation for the Advancement of Military Medicine, Inc., 6720-A Rockledge Drive, Bethesda, MD 20817 USA; 3 Blast Induced Neurotrauma Branch, Center for Military Psychiatry and Neurosciences, Walter Reed Army Institute of Research, 503 Robert Grant Ave, Silver Spring, MD 20910 USA

**Keywords:** Neuroscience, Brain injuries, Biomedical engineering

## Abstract

Animal studies provide valuable insights on how the interaction of blast waves with the head may injure the brain. However, there is no acceptable methodology to scale the findings from animals to humans. Here, we propose an experimental/computational approach to project observed blast-induced molecular changes in the rat brain to the human brain. Using a shock tube, we exposed rats to a range of blast overpressures (BOPs) and used a high-fidelity computational model of a rat head to correlate predicted biomechanical responses with measured changes in glial fibrillary acidic protein (GFAP) in rat brain tissues. Our analyses revealed correlates between model-predicted strain rate and measured GFAP changes in three brain regions. Using these correlates and a high-fidelity computational model of a human head, we determined the equivalent BOPs in rats and in humans that induced similar strain rates across the two species. We used the equivalent BOPs to project the measured GFAP changes in the rat brain to the human. Our results suggest that, relative to the rat, the human requires an exposure to a blast wave of a higher magnitude to elicit similar brain-tissue responses. Our proposed methodology could assist in the development of safety guidelines for blast exposure.

## Introduction

Exposure to explosion-induced blast waves presumably causes mild traumatic brain injury (TBI) in humans, leading to acute and chronic neurological disorders, such as impaired memory or concentration^1–3^. Indeed, different studies provide compelling evidence that connects a history of non-impact, blast-wave exposure in military settings with these adverse neurobehavioral conditions^4–7^, highlighting the need to properly assess and characterize the effects of blast loads to the human head. While multiple animal-model studies of blast-induced neurotrauma have contributed to our understanding of this phenomenon^8–11^, we still lack a valid methodology to project (i.e., to scale) these findings from animals to humans, which would ultimately help in the development of safety guidelines and injury thresholds for blast exposure.

Several approaches have been proposed to project injuries observed in an animal’s brain to the human brain. For example, mass-based scaling, initially conceptualized to project blast-induced lung pathology in animals to humans^12,13^, uses either the animal’s body mass or brain mass to estimate the duration of exposure that causes similar brain injury in humans^14^. In particular, this scaling methodology has been implemented to infer gross brain injury in humans from rodent, rabbit, and ferret models^15–18^. However, the mass-based approach restricts scaling to the duration of the blast-overpressure (BOP) exposure without considering its intensity. In addition, it neglects the differences in head anatomy and brain geometry between species, which are known to influence the transmission of blast waves through the brain tissues and are likely to affect the estimated injury threshold of each species^14,19^. Alternatively, mathematical models that predict biomechanical responses, such as intracranial pressure, stress, and strain, have also been proposed to infer brain injury in humans from observations in animals exposed to blunt or blast loads^14,19–23^. Ideally, such a biomechanical-based approach consists of establishing correlates in an animal model between observed brain injuries and the associated predicted biomechanical responses, and developing equivalence relationships to project the injuries across species^14,19–23^. Provided that the mathematical model has high fidelity, biomechanical-based scaling should inherently account for interspecies differences not only in head mass but also in head anatomy, brain geometry, and tissue material properties, which are not considered in mass-based scaling^14,15,19,24^.

Recently, a few studies have used biomechanical-based scaling laws to project brain injuries resulting from blunt impact to the head across species^19–23^. However, only a couple of studies have used this approach to infer the effect of blast insults to the human head^25,26^. For example, Jean et al. theorized a scaling law as a function of model-predicted intracranial pressure and material properties of the facial flesh, skull, and brain^25^. Using this scaling law, they determined that the human brain has a lower tolerance to blast exposure compared to mice and pigs. In a different study, Saunders et al. estimated the blast loads that cause similar brain injuries in humans and pigs by comparing predicted blast-induced intracranial pressures with observed injury thresholds for impact-induced concussion^26^. Their results indicated that to elicit the same extent of brain injury in the human as in the pig, the human requires a lower blast-load exposure. While these efforts advanced our understanding of how to implement mechanics-based principles to relate blast-induced brain injuries across species, it is not clear whether and to what extent the predicted biomechanical responses correlate with the observed brain injuries. In addition, it is not known whether these results would hold had the models represented the detailed cerebrovascular network and used species-specific brain material properties, which are necessary to enhance model fidelity to blast loads^27–29^.

Recently, our team developed and validated three-dimensional (3-D), high-fidelity, finite-element (FE) models of a rat head^27,30^ and a human head^28^ to simulate BOP exposure and predict the resulting biomechanical responses of the brain tissues. In the rat FE model^27,30^, we represented the detailed anatomy of the brain, including a comprehensive network of the cerebral vasculature, while using rat-specific material properties characteristic of the high strain rates observed in blast exposures, which we obtained through a separate study^31^. Compared with lower-fidelity models, our high-fidelity rat-head model predicted lower brain-tissue strains by as much as 33%^27^. Similarly, in the human-head FE model^28^, we represented the brain-surface convolutions, a detailed network of cerebral veins and arteries, and non-linear brain-tissue properties to enhance model fidelity. Here again we showed that the incorporation of these model enhancements redistributed the peak brain-tissue strains by as much as 30% and yielded peak strain differences of up to 7% when compared to lower-fidelity models^28^. Taken together, these recent efforts emphasize the importance of high-fidelity modeling to more comprehensively account for the biomechanical responses induced by blast exposure.

In the present study, we developed a new biomechanical-based approach to project blast-induced molecular changes in the rat brain to the human brain, with the underlying assumption that protein changes are associated with potential brain injuries. In particular, we conducted shock-tube experiments on rats and used a high-fidelity mathematical FE model of the rat brain to establish correlates between observed protein changes and predicted BOP-induced biomechanical responses. Next, using the identified correlates for the rat and a similar set of predicted biomechanical responses for the human, we determined the incident BOPs that induced the same responses in the brain of each species. Using this equivalence in BOPs, we then computed rat-to-human BOP scaling factors and inferred the protein changes in the human brain from the corresponding observations in the rat. By knowing the BOP and the associated protein change in rats, we can multiply the BOP by the scaling factor to estimate the blast exposure in humans that would yield an equivalent protein change. We hypothesize that we can identify biomechanical responses that correlate with observed changes in the expression of putative protein brain markers of blast exposure in a rat model. We further hypothesize that by combining data from blast experimental studies and predictions from high-fidelity computational models, we are able to project protein changes in the rat brain to the human.

## Methods

### Overview

**Figure 1:**
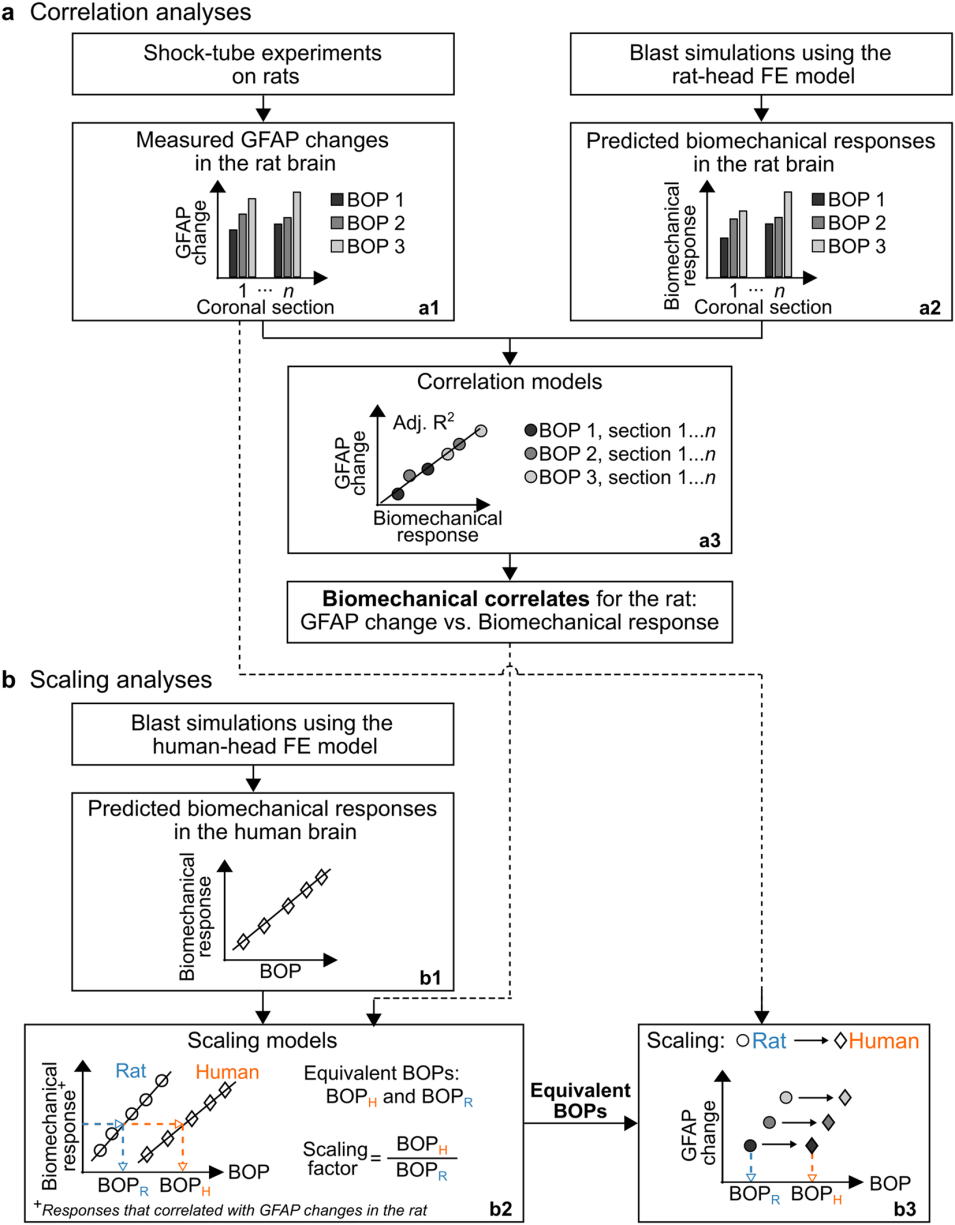
Flowchart of the biomechanical-based methodology to scale blast-induced molecular changes in the rat brain to the human brain. (**a**) Correlation analyses. We performed single shock-tube exposures on rats at different incident blast overpressures (BOPs) and characterized the brain-tissue changes in glial fibrillary acidic protein (GFAP) resulting from the blast-wave exposures (**a1**). Then, using a high-fidelity finite-element (FE) model of a rat head, we conducted blast simulations at the same BOPs as those of the experiments and computed the biomechanical responses (i.e., intracranial pressure, von Mises stress, maximum principal strain, and strain rate) in the rat brain (**a2**). Using the experimental and simulation data, we developed correlation models to identify correlates between the biomechanical responses and GFAP changes (**a3**). We performed these analyses for three brain regions: corpus callosum, hippocampus, and brainstem. (**b**) Scaling analyses. Using a high-fidelity FE model of a human head, we performed blast simulations at different BOPs and computed the biomechanical responses in the brain tissues (**b1**). Next, using the biomechanical correlates for the rat and the predicted responses for the human, we developed scaling models and determined equivalent BOPs in rats and humans that induced similar biomechanical responses in a given brain region of the two species (**b2**). In addition, using the equivalent BOPs, we computed the corresponding rat-to-human BOP scaling factors as the ratio of the equivalent BOPs (**b2**) and projected the experimentally measured GFAP changes in the rat brain to the human (**b3**). *Adj. R*^*2*^ adjusted R-squared, *BOP*_*H*_ equivalent blast overpressure in the human, *BOP*_*R*_ equivalent blast overpressure in the rat.

We used a two-step biomechanical-based methodology, which combined experimentation and computation, to scale blast-induced molecular changes in the rat brain to the human brain. In the first step (Fig. 1a), which we referred to as correlation analyses, we matched experimentally observed protein changes in the brain of a rat resulting from a single blast-wave exposure at different BOPs (Fig. 1a,a1) to computationally predicted biomechanical responses for the same exposures (Fig. 1a,a2). We identified matches (i.e., correlates) for three brain regions: corpus callosum, hippocampus, and brainstem (Fig. 1a,a3). In the second step (Fig. 1b), which we termed scaling analyses, we used the correlates for the rat and computationally predicted biomechanical responses of human-brain tissue for different blast exposures (Fig. 1b,b1) to determine equivalent BOPs that elicited responses in the human brain similar to those predicted in the rat brain. To this end, for each brain region, we used linear regression curves to match biomechanical responses across the two species (Fig. 1b,b2). Finally, we used this equivalence to compute scaling factors (Fig. 1b,b2) and project the measured protein changes in the rat brain to the human brain (Fig. 1b,b3). The key underlying assumption of our approach is that the same biomechanical response in the brain tissue of the two species yields the same protein changes in the two species.

### Correlation analyses

#### Shock-tube experiments on rats

To characterize changes in the brain of a rat resulting from blast exposures, we performed shock-tube experiments at targeted incident BOPs of 80, 100, or 130 kPa on 10- to 12-week-old (330–350 g) male Sprague–Dawley rats (Charles River Laboratories, Wilmington, MA). The Animal Care and Use Review Office of the U.S. Army Medical Research and Development Command, Fort Detrick, MD, as well as the Institutional Animal Care and Use Committee at the Walter Reed Army Institute of Research (WRAIR), Silver Spring, MD, approved all experimental protocols. We conducted all experiments in compliance with the Animal Research: Reporting of In Vivo Experiments guidelines.

To conduct the shock-tube experiments, we used an Advanced Blast Simulator (ABS) located at WRAIR and placed the animals inside the tube in a head-only-exposure configuration, as described in our previous studies^30,32^. Briefly, in this configuration, we limited the blast exposure exclusively to the animal’s head by keeping the torso outside of the shock tube, while securing the head inside the tube’s test section. In particular, we secured the torso of the animal to a vertical silicone cylinder outside of the shock tube, and arranged for the head to project into the test section through an opening in the bottom wall. In addition, we wrapped flexible strings around the head and attached them to two vertical pins to keep the head in a vertical orientation without restricting its motion. Moreover, we aligned the anterior–posterior planes of the rat perpendicular to the direction of the blast-wave propagation, with the ventral surface of the animal facing the blast wave^30,32^.

We arbitrarily assigned rats to control or head-only-exposure groups. Based on a previous power calculation, we determined that a sample size of n = 4 was sufficient to observe a statistically significant difference (*p* < 0.05) in protein levels between groups with a statistical power of 0.80 (effect size = 1.87)^32^. Hence, we assigned rats to control (n = 5 for 80 kPa, n = 5 for 100 kPa, and n = 4 for 130 kPa BOP) and head-only-exposure groups (n = 5 for 80 kPa, n = 10 for 100 kPa, and n = 10 for 130 kPa BOP). To conduct the experiments, we anesthetized the animals by placing them inside an induction chamber and administering 4% isoflurane for 6 min. Then, using the experimental setup described above, we exposed the rats to a single blast. Control animals received the same treatment, except for the exposure to the blast wave. During the experiments, we measured the static pressure–time profile of the incident blast wave at a location inside the test section of the shock tube using a custom-made pencil probe, as described in Rubio et al.^32^. In addition, we visualized the movement of the head at 25,000 frames per second using a high-speed camera (Phantom model v1212; Vision Research Inc., Wayne, NJ).

#### Assessment of GFAP changes in the rat brain

Following the protocol described in our previous study^32^, we conducted immunohistochemical analyses on brains harvested from control and head-only-exposed rats to assess changes in brain tissues resulting from the blast exposure. In particular, we assessed the expression of glial fibrillary acidic protein (GFAP), i.e., a biomarker of astrocytes, at 24 h after blast exposure (Fig. 1a,a1). To this end, we anesthetized the rats with 5% isoflurane for at least 8 min and transcardially perfused them with phosphate-buffered saline, followed by 4% paraformaldehyde acid (PFA) to fix the brains. After collecting the brains from the cranial vaults, we post-fixed them in 30% sucrose for 24 h and stored them in 4% PFA. We shipped all the fixed brains to FD NeuroTechnologies (Columbia, MD), where they were cut into coronal sections using a cryostat and processed for immunohistochemistry using primary antibodies (#556330; BD Biosciences, San Jose, CA; 1:350 dilution) and secondary antibodies with a fluorescent tag to detect GFAP (#A21202; Thermo Fisher Scientific, Waltham, MA; 1:250 dilution). In particular, the coronal sections were incubated with primary antibody diluted in 0.3% Triton X-100 solution and 2% blocking serum at 4 °C for 24 h, followed by incubation with secondary antibody diluted in a similar solution at room temperature for 1 h. As detailed in Rubio et al.^32^, we performed these analyses on each of 12 coronal brain sections (30 µm thick), which were serially cut from − 1 to − 12 mm relative to Bregma.

To quantify GFAP changes in the rat brain resulting from the head-only exposure, we digitized (10 × magnification) each stained coronal brain section harvested from the control and blast-exposed rats. As described in our previous study^32^, this magnification provided enough resolution to analyze the distribution and expression of positively stained cells in the main brain regions within a coronal section. Then, as detailed in Supplementary Fig. S1, we used rat-brain atlases^33,34^ as guides and the ImageJ software (National Institutes of Health, Bethesda, MD) to quantify the total intensity (i.e., the integrated density) of GFAP expression in three brain regions: corpus callosum, hippocampus, and brainstem. We analyzed these brain regions because they regulate cognitive and motor functions affected by blast exposure^35–38^. To identify changes in GFAP between control and head-only-exposed rats, we compared the data for a given brain region across each coronal section using the Mann–Whitney test, with a significance criterion of *p* < 0.05. We conducted all statistical analyses using the statistical software R^39^.

#### Blast simulations using a finite-element model of a rat head

**Figure 2:**
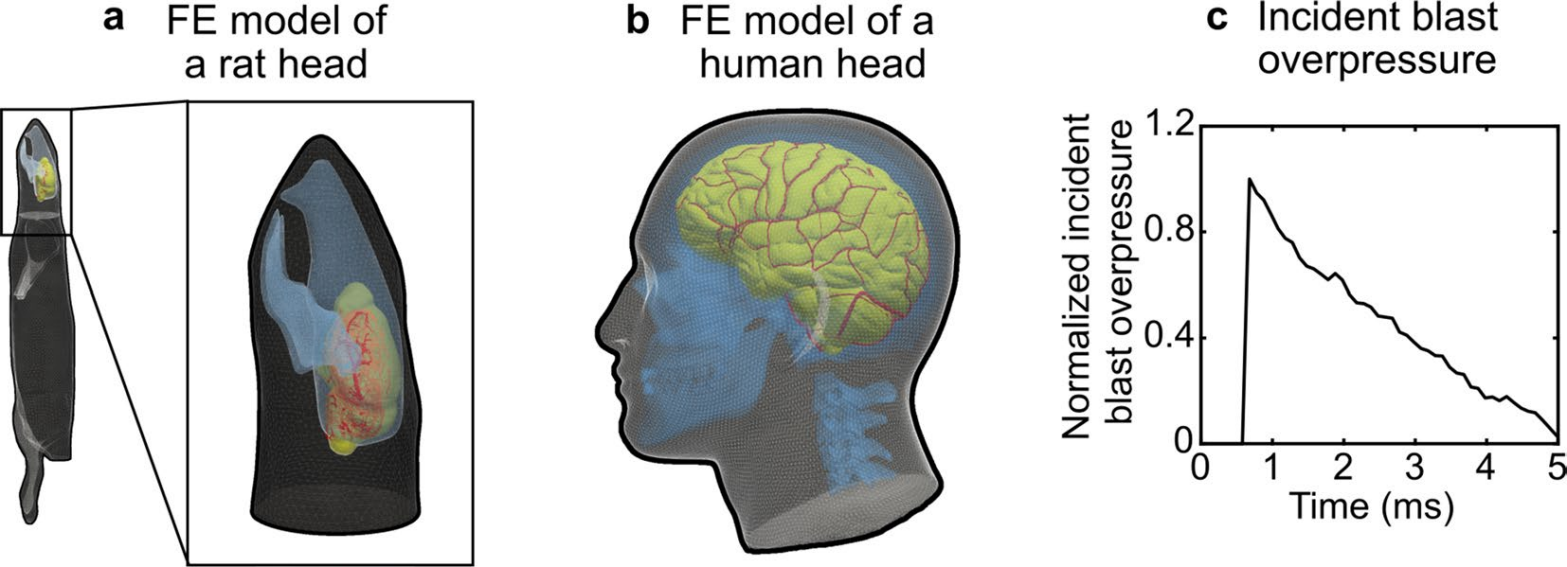
Representation of the previously validated high-fidelity, three-dimensional finite-element (FE) model of (**a**) a rat head^27,30^ and (**b**) a human head^28^ used in the blast simulations. (**c**) Normalized pressure–time profile of the incident blast overpressure (BOP) used as the input to the blast simulations. To perform the simulations, we scaled the amplitude of this normalized profile by multiplying it by the target BOP magnitude.

We previously developed and validated a high-fidelity, 3-D FE model of a rat head to simulate exposure to blast waves in a shock tube^27,30^ (Fig. 2a). Briefly, in this model, we represented the skin, face muscles, skull, brain, and torso of a rat using quadratic tetrahedral (C3D10M) volume meshes with an average element size of 0.24 mm (total number: 268,741). In addition, we represented a comprehensive network of cerebral vasculature using reduced-integration (S3R) shell elements with an average element size of 0.07 mm (total number: 316,182). We previously determined these mesh parameters using mesh-sensitivity analyses^27,30^. For the brain and the cerebral vasculature, we used rat-specific material properties representative of the high strain rates typical of blast exposures^30^.

To simulate a head-only exposure in a shock tube, we developed a 3-D FE model of a partial, 1.50-m-long rectangular shock tube with a square cross-sectional area of 0.60 m × 0.60 m. We meshed the shock tube using approximately 1 million Eulerian (EC3D8R) elements and assigned the properties of air as an ideal gas^27,30^. As in our previous work^27,30^, we used the coupled Eulerian–Lagrangian technique in ABAQUS (Dassault Systèmes, Vélizy-Villacoublay, France) to couple the shock-tube FE model with the rat FE model, and used the pressure–time profile of the incident BOP (Fig. 2c) to drive the simulations. In addition, we positioned the rat in the same orientation relative to the shock-front propagation, as described above in the shock-tube experiments.

Using the coupled model, we performed simulations of a single blast exposure at the same incident BOPs as in the shock-tube experiments (i.e., 80, 100, or 130 kPa). From the simulations, we computed four biomechanical responses throughout the rat brain: intracranial pressure (ICP), strain rate (SR), von Mises stress (VMS), and maximum principal strain (MPS) (Fig. 1a,a2). As previously detailed^28,40^, these responses have been associated with different neurological conditions, including concussion^1^, axonal stretching^41^, white-matter injury^42^, and neuronal injury^43^. Because our goal was to investigate the biomechanical responses in the brain solely due to the initial interaction of the blast wave with the head^41^, we conducted all simulations for a duration of 5 ms, which corresponded to the initial timeframe taken by the incident pressure wave to traverse the rat head. We performed all simulations using ABAQUS/Explicit on a SGI ICE XA system configured with Intel E5-2698v4 Broadwell processors. Using 80 CPU cores and a stable time step of 95 ns determined by the double-precision ABAQUS solver, we completed each 5-ms simulation in 6 h.

We further post-processed the simulation data by extracting region-specific (i.e., corpus callosum, hippocampus, and brainstem) biomechanical responses. To this end, we developed an in-house module using a customized Python script^44^ and the software 3D Slicer^45^. Briefly, using rat-brain atlases^33,34^, we identified representative images of the same 12 coronal brain sections used in the immunohistochemical analyses. Then, we mapped these atlas images into the corresponding coronal sections in the rat FE model using a curvature-based contour-matching algorithm^46^ followed by a smoothing transformation. Using the mapped atlas images, we delineated the different brain regions in the rat FE model by selecting the elements within each region. Finally, we extracted region-specific biomechanical responses for the entire simulation time using the data from each of the elements within a region (Fig. 1a,a2).

#### Identification of biomechanical correlates for the rat

We developed linear-regression models, henceforth referred to as “correlation models,” to evaluate the degree of association between the FE model-predicted biomechanical responses and the experimentally measured GFAP changes in the rat brain resulting from a head-only exposure (Fig. 1a,a3). To this end, using the immunohistochemical data for each brain region within a coronal section, we computed a GFAP ratio by dividing the corresponding mean value of the head-only-exposed rats by that of the controls. In addition, using the simulation data for each brain region within a coronal section, we determined the peak 90th percentile of each biomechanical response over the entire simulation time. To formulate the correlation models, we considered the GFAP ratio as the dependent variable and the incident BOP (either its peak pressure or impulse) and the biomechanical response as the independent variables. Then, we developed the correlation models by fixing the incident BOP, while varying the biomechanical response (i.e., the second independent variable). Using this procedure, we analyzed a total of 24 (8 coronal sections × 3 BOPs) correlations per biomechanical response and brain region (Fig. 1a,a3). Finally, we assessed the correlation between the measured GFAP changes and a predicted biomechanical response by computing the adjusted R-squared (Adj. R^2^) value of the associated regression model. We developed all correlation models using the statistical software R^39^.

### Scaling analyses

#### Blast simulations using a finite-element model of a human head

Recently, our team developed and validated a high-fidelity, 3-D FE model of a 50th percentile U.S. male head to simulate blast exposure in a laboratory shock tube^28^ (Fig. 2b). Briefly, the model of the human head consists of the skin, adipose tissue, eyes, sinuses, cervical spine, skull, brain, meninges, and a detailed network of cerebral arteries and veins. We represented all components, except for the cerebral vasculature, using modified quadratic tetrahedral (C3D10M) volume meshes with an average element size of 2.3 mm (total number: 4,289,775). In addition, we represented the cerebrovascular network using reduced-integration (S3R) shell elements having an average element size of 0.27 mm (total number: 825,898). We previously determined the optimal mesh parameters using mesh-sensitivity analyses^28,40^. Moreover, we implemented non-linear brain-tissue properties as described by Subramaniam et al.^28^.

To simulate blast exposures in a shock tube, we coupled the human-head FE model with the same 3-D FE model of a partial shock tube described above for the blast simulations with the rat FE model. In addition, we aligned the anterior–posterior planes of the human head in the direction of the blast-wave propagation, with the head facing the blast wave. As previously detailed^28^, we coupled the shock-tube FE model with the human-head FE model using the coupled Eulerian–Lagrangian technique in ABAQUS. Using the coupled model, we performed 5-ms simulations of a single blast exposure at several incident overpressures (BOPs: 13, 25, 50, 80, 150, 180, or 200 kPa). We chose this spectrum of BOPs to encompass the range of pressures in the rat exposures. We conducted all simulations using ABAQUS/Explicit on a HPE SGI 8600 system configured with Intel 8168 Skylake processors. Using 96 CPU cores and a stable time step of 21 ns determined by the ABAQUS solver, we completed each 5-ms simulation in 17 h. Finally, from the blast simulations, we extracted region-specific biomechanical responses using the in-house 3D Slicer module described above, except that here we used a human-brain atlas^47^ as a reference to delineate the corresponding brain regions (Fig. 1b,b1).

#### Calculation of equivalent BOPs and rat-to-human scaling factors

We developed linear-regression models, henceforth referred to as “scaling models,” to determine equivalent incident BOPs that induced biomechanical responses in the human brain similar to those predicted in the rat brain (Fig. 1b,b2). In particular, for each brain region, we developed separate scaling models for the rat and the human, considering only the biomechanical responses identified in our correlation analyses for rats. To this end, first, we utilized the data from the seven blast-exposure simulations for the human-head FE model. Similarly, for the rat, we used the data from the three simulations detailed above (BOPs: 80, 100 and 130 kPa) plus six additional simulations (BOPs: 13, 25, 50, 150, 180, and 200 kPa) to obtain a better regression fit. Second, as in our correlation analyses, we used the simulation data for each brain region within a coronal section to compute the peak 90th percentile of each biomechanical response over the entire simulation time. Third, for each of the two species, we computed a *per-BOP* estimate of the biomechanical response by averaging the corresponding values of all the coronal sections for a given brain region. Finally, using these per-BOP estimates, we developed scaling models by considering the biomechanical response as the dependent variable and the incident BOP as the independent variable. We developed all scaling models using the statistical software R^39^.

For each brain region, we utilized the scaling models to determine the equivalent BOPs that induced the same biomechanical response in rats and humans (Fig. 1b,b2). To this end, we intersected each species-specific scaling model, represented by its respective regression curve, with a target biomechanical response value on the y-axis, and determined the equivalent BOPs on the x-axis (Fig. 1b,b2). For each brain region and biomechanical response, we repeated this calculation three times, using as a target value the per-BOP estimate from the rat simulations at incident BOPs of 80, 100, and 130 kPa. Finally, for each brain region, we computed the rat-to-human BOP scaling factor by taking the ratio of the three pairs of equivalent BOPs. Given a BOP that causes a GFAP change in the rat brain, we can multiply the rat BOP by the scaling factor to estimate the BOP that would induce the same GFAP change in the human brain (Fig. 1b,b3).

Following this procedure, we projected our experimentally measured blast-induced GFAP changes in the rat brain to the human (Fig. 1b,b3). For this purpose, using the GFAP data for a given BOP described above in the correlation analyses, we computed a *per-BOP* GFAP ratio by averaging the respective values of all the coronal sections for a given brain region. Then, we projected this per-BOP GFAP ratio from the rat to the human by matching it with the respective equivalent BOPs (Fig. 1b,b3). We repeated this procedure for each of the three BOPs in the rat-exposure experiments. This interspecies projection hinges on the *equivalence assumption*, which states that identical biomechanical responses cause equivalent brain injuries across species^14,22,24^. This assumption was based on the work of Wu et al., who provided compelling evidence that similar biomechanical responses to blunt impact to the head cause equivalent TBI across different species (i.e., human, pig, and macaque)^22^.

### Ethics approval

In conducting research using animals, the investigators adhered to the Animal Welfare Act Regulations and other Federal statutes relating to animals and experiments involving animals and the principles set forth in the current version of the Guide for Care and Use of Laboratory Animals, National Research Council.

## Results

### Correlation analyses

#### Shock-tube experiments on rats

We conducted head-only exposures on rats at targeted incident BOPs of 80, 100, and 130 kPa. The measured peak overpressures were 81 ± 5 kPa, 95 ± 2 kPa, and 128 ± 19 kPa (mean ± one standard deviation), respectively. Supplementary Table S1 describes other relevant blast-wave parameters, such as impulse and duration. In addition, the incident pressure–time profiles from the shock-tube experiments showed a nearly instantaneous rise to the peak overpressure, followed by a rapid nonlinear decay (Supplementary Fig. S2). Based on a quantitative analysis of high-speed video footage and a digital marker on the animal’s nose, a common landmark used for head-motion tracking^50^, we observed minimal head movement during the initial 5 ms after exposure to an 130 kPa BOP [~ 0.7 mm along the direction of shock propagation (ventral-dorsal plane) and ~ 7.0 mm perpendicular to it (anterior–posterior plane)]. This suggests that the exposure resulted in minimum neck dynamics, even for the highest BOP.

#### Blast-induced GFAP changes in the rat brain

**Figure 3:**
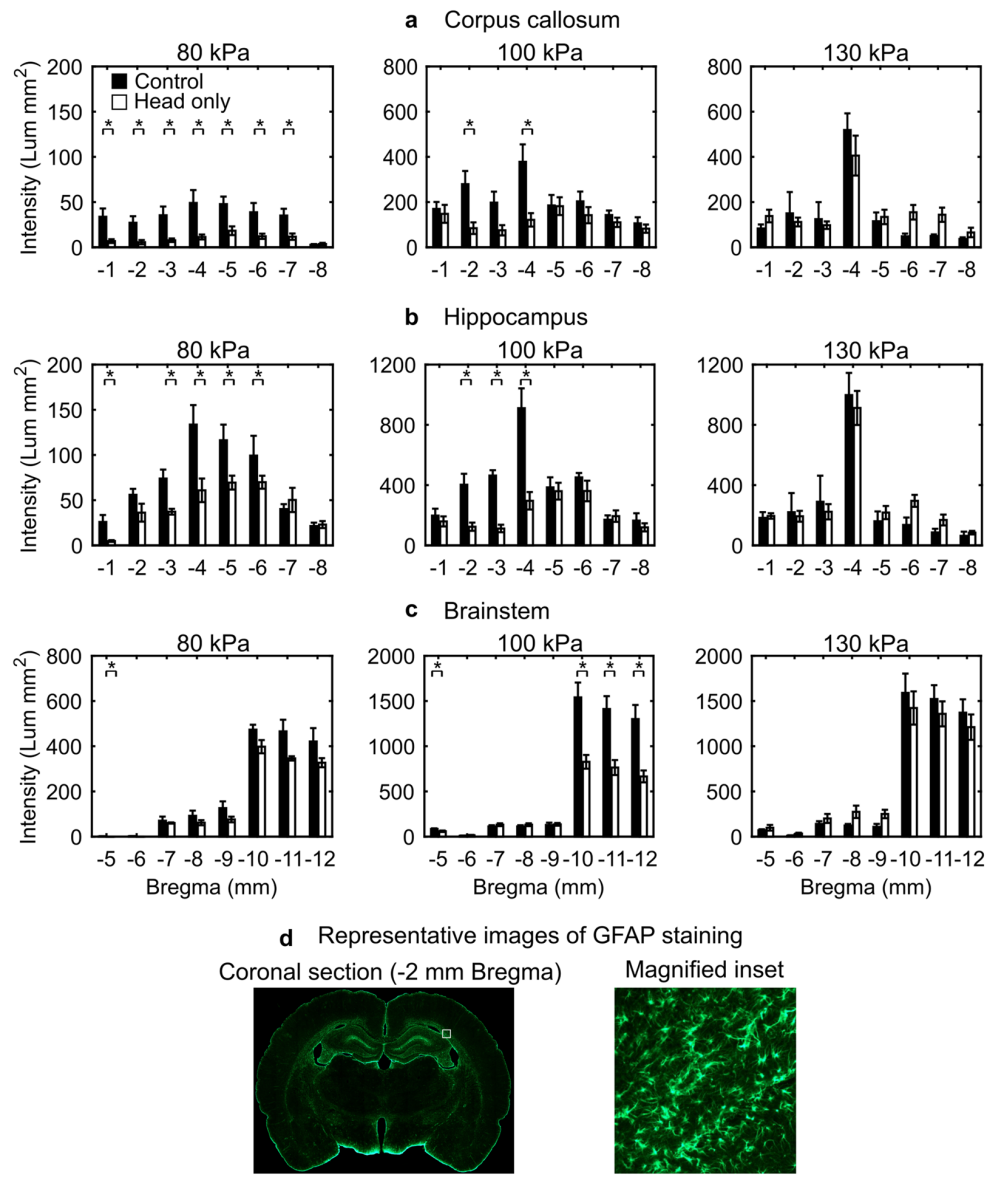
GFAP-positive staining in the (**a**) corpus callosum, (**b**) hippocampus, and (**c**) brainstem resulting from a single head-only blast-wave exposure in rats for incident blast overpressures (BOPs) of 80, 100, and 130 kPa in a shock tube. We conducted immunohistochemical analyses (i.e., GFAP staining) on coronal brain sections serially cut from − 1 to − 12 mm relative to Bregma and harvested them at 24 h post-exposure. To identify changes in GFAP-positive staining between control and head-only-exposed rats, we compared the data for a given brain region across each coronal section using the Mann–Whitney test. Asterisks denote statistically significant differences (*p* < 0.05) between control (n = 5 for 80 and 100 kPa, and n = 4 for 130 kPa BOP) and blast-exposed groups (n = 5 for 80 kPa, and n = 10 for 100 and 130 kPa BOP). The bar height and vertical line length represent the mean and one standard error of the mean, respectively. The corpus callosum and hippocampus spanned from − 1 to − 8 mm relative to Bregma, while the brainstem spanned from − 5 to − 12 mm relative to Bregma. For the 80-kPa plots, we reduced the y-axis scale for each brain region to better illustrate the data. (**d**) Representative images of GFAP staining in a coronal brain section located at − 2 mm relative to Bregma. The magnified inset shows positively stained cells in the coronal section. *GFAP* glial fibrillary acidic protein.

We performed immunohistochemical analyses to assess changes in GFAP in the rat brain resulting from a head-only exposure. Based on the power calculation described in the Methods (n = 4 per group), we targeted a sample size of n = 5 per group, in case we needed to exclude an animal from the analyses because of problems while processing the brain tissues. In fact, due to processing artifacts, we removed one control animal from the 130 kPa group. In addition, for the 100 kPa and 130 kPa blast-exposed groups, we increased the sample size to n = 10 per group to confirm the trends of the results. The inclusion of these additional animals did not change the trends or the statistical significance of the results, hence, we included them in our analyses. Moreover, to minimize the possibility of batch artifacts confounding our results, we conducted the staining in three separate batches, where in each batch we included animals exposed to one of three BOPs and its corresponding control group to compare against each other. Figure 3d shows representative images of GFAP staining in a coronal brain section.

Relative to controls, GFAP-positive staining consistently decreased (*p* < 0.05) in the corpus callosum of rats exposed to a blast wave of 80 kPa (from − 1 to − 7 mm relative to Bregma; Fig. 3a) and 100 kPa (at − 2 and − 4 mm relative to Bregma; Fig. 3a). In contrast, albeit not statistically significant when compared to controls, we observed bidirectional changes in GFAP-positive staining for rats exposed to a blast wave of 130 kPa (Fig. 3a).

When compared to controls, GFAP-positive staining decreased (*p* < 0.05) in the hippocampus of rats exposed to an incident wave of 80 kPa (at − 1 and from − 3 to − 6 mm relative to Bregma; Fig. 3b) and 100 kPa (from − 2 to − 4 mm relative to Bregma; Fig. 3b). Although not statistically significant when compared to controls, GFAP-positive staining decreased (from − 2 to − 4 mm relative to Bregma; Fig. 3b) and increased (at − 1 and from − 5 to − 8 mm relative to Bregma; Fig. 3b) for rats exposed to an incident BOP of 130 kPa.

Relative to controls, GFAP-positive staining consistently decreased (*p* < 0.05) in the brainstem of rats exposed to a blast wave of 80 kPa (at − 5 mm relative to Bregma; Fig. 3c) and 100 kPa (at − 5 and from − 10 to − 12 mm relative to Bregma; Fig. 3c). In addition, although not statistically significant when compared to controls, we observed bidirectional changes in GFAP-positive staining for rats exposed to an incident wave of 130 kPa (Fig. 3c).

#### Biomechanical responses of the rat brain due to blast exposure

**Figure 4:**
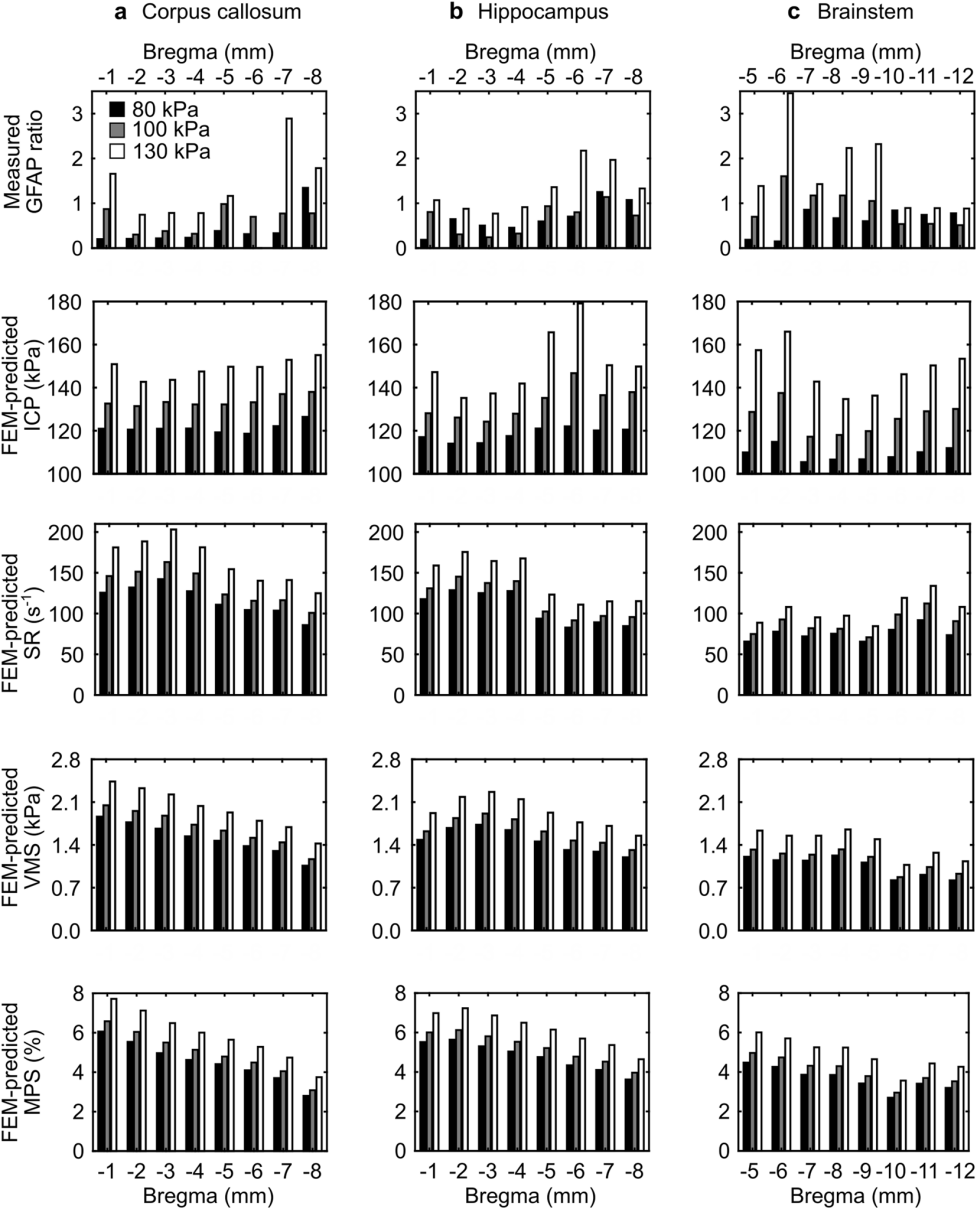
Experimentally measured changes in GFAP and computationally predicted biomechanical responses in the (**a**) corpus callosum, (**b**) hippocampus, and (**c**) brainstem of a rat resulting from a single head-only blast-wave exposure for three blast overpressures. Using the immunohistochemical data for each brain region within a coronal section, we computed a GFAP ratio by dividing the corresponding mean value of the head-only-exposed rats by those of the controls. Using the blast-simulation data for each brain region within a coronal section, we determined the peak 90th percentile of each biomechanical response over the entire simulation time of 5 ms. The corpus callosum and hippocampus spanned from − 1 to − 8 mm relative to Bregma, while the brainstem spanned from − 5 to − 12 mm relative to Bregma. *FEM* finite-element model, *GFAP* glial fibrillary acidic protein, *ICP* intracranial pressure, *MPS* maximum principal strain, *SR* strain rate, *VMS* von Mises stress.

From the simulations using the coupled FE model of the shock tube and the rat head, we determined the biomechanical responses (i.e., ICP, SR, VMS, and MPS) of the rat brain for the same three BOPs. For the 80 kPa and 100 kPa BOPs, we observed lower ICP values in the brainstem compared to the corpus callosum and hippocampus (Fig. 4 and Supplementary Fig. S3). For these BOPs, the peak 90th percentile ICP values ranged from 119 to 139 kPa in the corpus callosum, from 114 to 147 kPa in the hippocampus, and from 105 to 138 kPa in the brainstem. For the 130 kPa BOP, the ICP values showed no obvious trend when compared between brain regions. For the three BOPs, we observed lower SR values in the brainstem compared to the corpus callosum and hippocampus (Fig. 4 and Supplementary Fig. S3). The peak 90th percentile SR values ranged from 86 to 203 s^−1^ in the corpus callosum, from 83 to 176 s^−1^ in the hippocampus, and from 66 to 134 s^−1^ in the brainstem. In terms of the VMS, the brainstem elicited lower stresses compared to the corpus callosum and hippocampus (Fig. 4 and Supplementary Fig. S3). For the brainstem, the peak 90th percentile VMS values ranged from 0.8 to 1.6 kPa across the three BOPs, whereas for the corpus callosum and hippocampus, they ranged from 1.1 to 2.4 kPa. For the three BOPs, we observed lower MPS values in the brainstem compared to the corpus callosum and hippocampus (Fig. 4 and Supplementary Fig. S3). In particular, the peak 90th percentile MPS values ranged from 2.8 to 7.7% in the corpus callosum, from 3.6 to 7.2% in the hippocampus, and from 2.7 to 6.0% in the brainstem.

#### Biomechanical correlates for the rat

We developed correlation models to assess the correspondence between experimentally measured GFAP changes in rats and computationally predicted biomechanical responses (Table 1). While we formulated separate correlation models using either the peak incident BOP or its impulse as one of the independent variables, we found that, with a few exceptions, the models developed using the impulse did not yield correlates as strong as those obtained using the peak incident BOP (Supplementary Table S2). Henceforth, we only present and discuss the results of the correlation models developed using the peak incident BOP.

**Table 1:** Regression models to assess the correlation between experimentally observed changes in glial fibrillary acidic protein (GFAP) and computationally predicted biomechanical responses in the rat brain due to a single head-only blast-wave exposure.

Biomechanical response (BR)	Regression parameters				
	Adj. R^2^	BR		BOP	
		Coefficient	*p*-value	Coefficient	*p*-value
**Corpus callosum**					
SR	0.66	− 0.015	0.001	0.036	< 0.001
ICP	0.58	0.076	0.006	− 0.020	0.157
VMS	0.56	− 0.905	0.009	0.029	< 0.001
MPS	0.53	− 22.091	0.017	0.026	< 0.001
**Hippocampus**					
SR	0.72	− 0.014	< 0.001	0.023	< 0.001
ICP	0.55	0.025	0.002	− 0.002	0.713
VMS	0.65	− 1.342	< 0.001	0.026	< 0.001
MPS	0.60	− 34.537	0.001	0.023	< 0.001
**Brainstem**					
SR	0.47	− 0.015	0.033	0.025	< 0.001
ICP	0.46	− 0.027	0.046	0.036	0.002
VMS	0.47	1.074	0.027	0.009	0.103
MPS	0.38	16.863	0.248	0.013	0.031

*Adj. R*^*2*^ adjusted R-squared, *BOP* blast overpressure, *ICP* intracranial pressure, *MPS* maximum principal strain, *SR* strain rate, *VMS* von Mises stress.

When compared to the other correlation models, the SR consistently showed the highest adjusted R^2^ for all brain regions, i.e., 0.66 for the corpus callosum, 0.72 for the hippocampus, and 0.47 for the brainstem (Table 1). For the corpus callosum, the adjusted R^2^ for the ICP (0.58), VMS (0.56), and MPS (0.53) models each explained at least 53% of the variance in the dependent variable (Table 1). In addition, for the hippocampus, the adjusted R^2^ for the VMS, MPS, and ICP models ranged from 0.55 to 0.65 (Table 1). Finally, for the brainstem, we obtained adjusted R^2^ for the VMS (0.47), ICP (0.46), and MPS (0.38), where each model explained at least 38% of the variance in the dependent variable. Note that, relative to the other two brain regions, the brainstem consistently showed lower adjusted R^2^ (Table 1).

### Scaling analyses

#### Biomechanical responses of the human brain due to blast exposure

**Figure 5:**
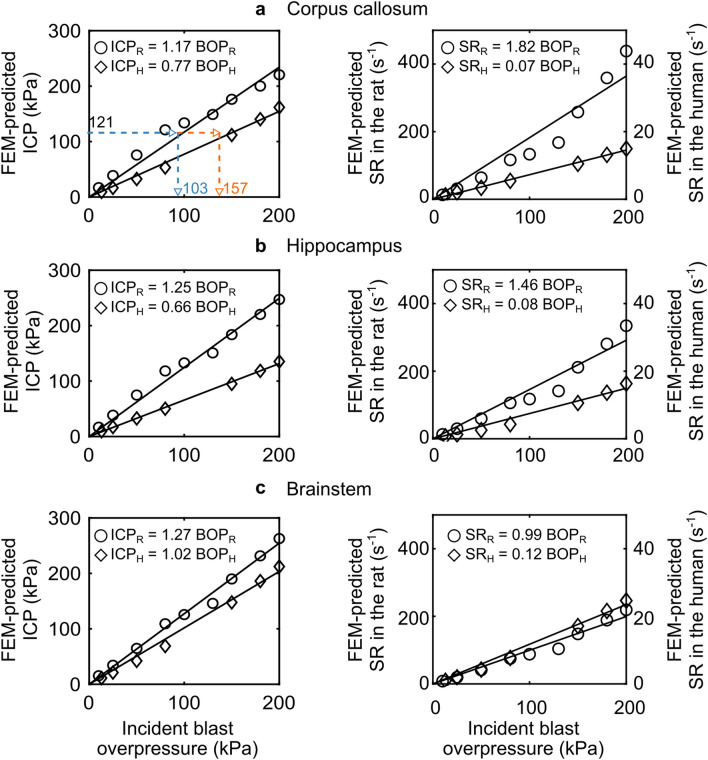
Scaling models of equivalent blast overpressures (BOPs) that elicited similar responses in the human brain and the rat brain. For a given biomechanical response value of strain rate (SR) or intracranial pressure (ICP) on the y-axis, the intercept with the two linear regression models provides the equivalent rat and human BOPs on the x-axis. To develop these scaling models, we only used biomechanical responses identified as correlates to GFAP changes in the rat. In these models, we considered the biomechanical correlates (i.e., SR and ICP) as the dependent variable and the incident BOP as the independent variable. The circles represent the rat data, while the diamonds show the human data. The solid lines represent the regression lines. In the SR graphs, we plotted the rat and human data on distinct y-axes. *BOP*_*H*_ equivalent blast overpressure in the human, *BOP*_*R*_ equivalent blast overpressure in the rat, *FEM* finite-element model, *ICP*_*H*_ intracranial pressure in the human, *ICP*_*R*_ intracranial pressure in the rat, *SR*_*H*_ strain rate in the human, *SR*_*R*_ strain rate in the rat.

From the simulations using the coupled FE model of the shock tube and the human head, we determined the biomechanical responses of the human brain for BOPs ranging from 13 to 200 kPa in a shock tube (Fig. 5). In particular, we focused our analysis on the SR because it was the highest correlate to GFAP changes for the rat (Table 1). We also analyzed the ICP because not only did we identify it as an adequate correlate, but because we previously used its predictions to validate our rat and human models against ICP measurements, obtaining a very good agreement^27,28,30^.

For all BOPs, we observed higher ICP values in the human brainstem compared to the corpus callosum and hippocampus (Fig. 5). Indeed, when we averaged over all coronal sections, the peak 90th percentile ICP ranged from 9 to 162 kPa in the corpus callosum (Fig. 5a), from 9 to 135 kPa in the hippocampus (Fig. 5b), and from 11 to 212 kPa in the brainstem (Fig. 5c). In terms of the SR, the human brainstem also elicited the highest values of the three regions. For the brainstem, the peak 90th percentile SR values ranged from 1 to 25 s^−1^ (Fig. 5c), whereas for the corpus callosum and hippocampus, they ranged from 1 to 16 s^−1^ (Fig. 5a,b).

#### Equivalent BOPs and rat-to-human scaling factors

Using the predicted ICP and SR, we developed scaling models to determine the equivalent BOPs that induced similar responses across species and the associated rat-to-human BOP scaling factors (Fig. 5 and Table 2). Below, we exemplify these calculations using the ICP scaling model for the corpus callosum (Fig. 5a, left, and Table 2, first row). First, we set a target ICP of 121.20 kPa, which we estimated by averaging the peak 90th percentile ICP values of the eight coronal sections from the rat corpus callosum (shown in Fig. 4), predicted for a BOP exposure of 80 kPa (i.e., our so-called *per-BOP* estimate). Next, we intersected each scaling model, represented by its respective regression curve, with this target ICP value on the y-axis [blue (rat) and orange (human) horizontal arrows, Fig. 5a, left], and determined the equivalent BOPs (103.59 kPa for the rat and 157.40 kPa for the human) on the x-axis. Finally, we computed the scaling factor of 1.52 for the corpus callosum by dividing the human equivalent BOP by that of the rat. Using the region-specific ICP scaling models, we repeated these calculations for the other two brain regions and determined scaling factors of 1.89 for the hippocampus and 1.25 for the brainstem (Table 2). Similarly, using the SR scaling models, we obtained the corresponding rat-to-human BOP scaling factors of 26.00 for the corpus callosum, 18.25 for the hippocampus, and 8.25 for the brainstem (Table 2).

**Table 2:** Measured changes in glial fibrillary acidic protein (GFAP), model-predicted biomechanical responses, equivalent incident blast overpressures (BOPs), and scaling factors, for three brain regions.

Experimental and simulated BOP	Measured GFAP ratio (mean ± SEM) in the rat^a^	Predicted ICP (kPa) in the rat^b^	Equivalent BOP (kPa) for ICP model^c^		Scaling factor for ICP model^d^	Predicted SR (s^−1^) in the rat^b^	Equivalent BOP (kPa) for SR model^c^		Scaling factor for SR model^d^
			Rat	Human			Rat	Human	
**Corpus callosum (n = 8 coronal sections, spanning from − 1 to − 8 mm relative to Bregma)**									
80 kPa	0.40 ± 0.14	121.20	103.59	157.40	1.52	116.39	63.95	1662.71	26.00
100 kPa	0.64 ± 0.09	133.74	114.31	173.69		133.20	73.19	1902.86	
130 kPa	1.40 ± 0.30	148.91	127.27	193.39		167.76	92.18	2396.57	
**Hippocampus (n = 8 coronal sections, spanning from − 1 to − 8 mm relative to Bregma)**									
80 kPa	0.68 ± 0.12	118.29	94.63	179.23	1.89	106.11	72.68	1326.38	18.25
100 kPa	0.66 ± 0.12	132.83	106.26	201.26		117.49	80.47	1468.63	
130 kPa	1.31 ± 0.18	150.83	120.66	228.53		141.33	96.80	1766.63	
**Brainstem (n = 8 coronal sections, spanning from − 5 to − 12 mm relative to Bregma)**									
80 kPa	0.60 ± 0.10	109.13	85.93	106.99	1.25	75.12	75.88	626.00	8.25
100 kPa	0.91 ± 0.14	125.73	99.00	123.26		87.90	88.79	732.50	
130 kPa	1.43 ± 0.23	145.87	114.86	143.01		103.86	104.91	865.50	

*BOP* blast overpressure, *GFAP* glial fibrillary acidic protein, *ICP* intracranial pressure, *SEM* one standard error of the mean, *SR* strain rate.

^a^Using the immunohistochemical data for each brain region within a coronal section, we determined a GFAP ratio by dividing the corresponding mean value of the head-only-exposed rats by those of the controls. Next, we computed a *per-BOP* estimate (shown in the table) by averaging the corresponding values of all the coronal sections for a given brain region.

^b^Using the rat simulation data for each brain region within a coronal section, we determined the peak 90th percentile of each biomechanical response over the 5-ms simulation. Then, we computed a *per-BOP* prediction (shown in the table) by averaging the corresponding values of all the coronal sections for a given brain region.

^c^Using the rat *per-BOP* predictions as target responses, we utilized the scaling models to determine the equivalent BOPs that elicited similar responses in both species.

^d^For each brain region and scaling model, we computed a scaling factor by taking the ratio of the equivalent BOPs.

## Discussion

We developed a biomechanical-based approach to project blast-induced molecular changes in the rat brain to the human brain (Fig. 1). To this end, we performed computational and experimental shock-tube studies on rats to identify correlates between model-predicted biomechanical responses and measured GFAP changes in brain tissues resulting from a single head-only exposure. Then, using these correlates and the model-predicted biomechanical response to blast in a human head, we determined the BOPs that induced equivalent brain responses across the two species. Finally, we utilized this equivalence in BOPs to obtain scaling factors and map measured GFAP changes in the rat to the human, for three brain regions. Given a rat BOP exposure and the associated observed GFAP change in the rat brain tissue, multiplying the BOP by the scaling factor provides the human BOP exposure that would yield an equivalent GFAP change in the human brain tissue.

We exposed rats to BOPs of 80, 100, or 130 kPa and assessed the resulting GFAP expression 24 h post-exposure in tissues of three brain regions, i.e., corpus callosum, hippocampus, and brainstem. Relative to controls, a single head-only blast-wave exposure caused bidirectional changes in GFAP in the rat brain. We observed significant decreases (*p* < 0.05) in GFAP levels for BOP exposures of 80 and 100 kPa, and modest, non-significant increases and decreases in exposures of 130 kPa (Fig. 3). Several studies, with BOPs ranging from 68 to 207 kPa, have reported elevated GFAP levels in different brain regions, such as hippocampus, corpus callosum, and amygdala, at 24 h and 7 days after whole-body^37,48,49^ and head-only^50,51^ rat exposures. These increases in GFAP expression have been associated with astrocyte reactivity, possibly aimed at providing neuroprotection and trophic support to the nervous tissue, as well as post-lesion regeneration of brain circuits^52–54^. In contrast, a few studies, with BOPs ranging from 100 to 207 kPa, have reported decreases in GFAP at 24 h and 7 days following rat exposure to a blast wave^32,50,51^. Interestingly, while different studies have reported opposite trends in GFAP changes^32,48,50,51^, only a handful have reported bidirectional changes^32,50,51^. These bidirectional changes suggest that the brain response could be influenced by different factors, such as BOP magnitude, degree of head motion, the complex wave-body interaction and concomitant load to the brain, and the presence of a negative-pressure phase in the incident blast wave^32,50,51^.

We observed differences in the blast-induced tissue responses between brain regions. For example, at − 5 mm relative to Bregma for an 80 kPa BOP, the measured GFAP ratio in the brainstem was as much as 67% and 104% smaller than those of the corpus callosum (0.19 vs. 0.38) and hippocampus (0.19 vs. 0.60), respectively (Fig. 4, first row). These differences could be associated with dissimilarities in white- and gray-matter content between regions, as well as the heterogeneous distribution of GFAP-expressive astrocytes in the rat brain^55–57^. Moreover, from the 130 kPa BOP simulations, at − 5 mm relative to Bregma, the predicted peak biomechanical responses were at least 5% (ICP), 32% (SR), 17% (VMS), and 2% (MPS) different in the brainstem as compared to the other two brain regions (Fig. 4). In addition to the distinct anatomical location of these regions, the inter-regional variability in biomechanical responses could be attributed to the unequal presence of vasculature and region-specific material properties assigned to the cerebrum, cerebellum, and brainstem in the FE model, which together influence and redistribute the brain-tissue responses to a blast insult^27–29^. Taken together, these regional disparities in molecular and biomechanical responses support the need for establishing *region-specific* correlates between experimentally measured GFAP changes and computationally predicted biomechanical responses, which we ultimately used to scale blast-induced changes between species.

Using experimental and computational studies, we identified the SR as the strongest biomechanical correlate of GFAP changes in each of the three regions of the rat brain (Table 1). In particular, when compared to the other biomechanical responses, the SR consistently correlated well with the measured GFAP changes in the rat brain, explaining as much as 72% of the variance in the blast-induced changes. Recently, two studies of the effect of blunt impact to the head also found evidence correlating SR and MPS responses to changes in brain tissues^22,23^. In particular, Donat et al. showed that model-predicted SR and MPS responses adequately correlate with changes in fractional anisotropy, expression of neurofilament protein, and microglia density in brain tissues of rats observed for up to 2 weeks after a blunt impact^23^. Separately, Wu et al. found that, for humans and pigs, model-predicted MPS and SR responses correlate well with observed mild TBI (i.e., concussion) resulting from a single blunt impact to the head, while only MPS responses satisfactorily estimate severe TBI (i.e., diffuse axonal injury and intracerebral hemorrhage) in the macaque^22^. Importantly, when Wu et al. combined their species-specific computations and observations and repeated the analyses, they obtained evidence that identical biomechanical responses cause similar TBI across the three species, providing evidence for the equivalence assumption, which is a fundamental assumption in our study.

Using the SR response for the rat and the equivalence assumption, we determined the incident overpressures that induced a similar SR response in the human and the associated rat-to-human BOP scaling factor for each of the three brain regions. In addition, we performed the same calculations for the ICP response because it yielded an adequate correlation coefficient (Adj. R^2^ ≥ 0.46) and it was the response used to validate our computational models^27,28,30^. For each response (SR and ICP), we obtained the smallest scaling factor in the brainstem (Table 2), indicating that, for a given BOP, the two species yield the most similar SR and ICP responses in this region of the brain (Table 2 and Fig. 5). In addition, when compared to the corpus callosum and the hippocampus, our results showed that the human brainstem required a lower-intensity BOP (e.g., for 80 kPa; ICP model: ~ 107 kPa vs. > 157 kPa; SR model: ~ 600 kPa vs. > 1300 kPa; Table 2) to elicit the same responses observed in the rat, suggesting that this brain region could be more susceptible to the incident wave, presumably due to its proximity to the skull and the potential reflections from the skin-skull, skull-subarachnoid space, and subarachnoid space-brain interfaces^28,58^.

The equivalent BOPs in humans estimated using the SR model correspond to very high levels of exposure (Table 2, second to last column). For reference, the classical *mass-based* scaling from Bowen^12,13,59^, which is formulated on *pulmonary injury* and scales *exposure duration* and *lethality*, estimates that a 5-ms exposure to a BOP >  ~ 690 kPa is potentially lethal to a 70-kg human. In our study, the equivalent BOPs in humans calculated with the SR model are elevated because we inferred them from relatively high levels of exposure on rats (> 80 kPa). However, because we determined the scaling factors using regression models fitted to data from a range of BOPs, we would like to emphasize that, once verified, our scaling methodology would be valid to determine equivalences for low as well as high BOP exposures.

Relative to the rat, the SR and ICP scaling factors consistently indicated that the human brain requires a higher BOP exposure to elicit similar biomechanical responses (Table 2). In contrast to these results, two studies found that the human brain is more vulnerable than the brain of mice^25^ and pigs^26^. Jean et al. developed a mouse-to-human scaling law using computationally predicted ICPs and a mathematical constant that accounts for the acoustic impedance of the brain and its surrounding structures (i.e., the skull and facial flesh)^25^. In addition to methodological and species (rat vs. mouse) differences, the discrepancy is mainly attributed to the large differences in ICP predictions in the human brain, which resulted from different selections of brain-tissue properties in the two computational models. For example, when we compared model-predicted peak ICP with experimental data for a BOP exposure of 100 kPa, our model yielded errors of 11% at the frontal lobe (135 kPa vs. 152 kPa) and 61% at the ventricle (65 kPa vs. 41 kPa)^28^. In contrast, Jean et al. predicted one peak ICP of 1,219 kPa in the brain, which was more than 700% larger than the measurements^25^. Separately, Saunders et al. developed mathematical expressions to project blast-induced mild TBI from the Yucatan minipig to the human^26^. In particular, using FE model-predicted ICPs and previously reported thresholds for blunt-induced mild TBI, they determined that the human requires a lower-magnitude BOP exposure to elicit a similar brain injury as that observed in the minipig. The fact that we observed the opposite when we compared humans and rats is not surprising because in addition to the numerous methodological differences (e.g., Saunders et al. used a simplified boundary condition and the same material properties for both species), there are distinct anatomical differences between Yucatan minipigs and humans (e.g., skull thickness of ~ 6.9 mm for humans^60^ vs. ~ 8.6 mm for Yucatan minipigs^29^).

A few experimental studies on rats have associated the SR resulting from a mechanical insult with different neuronal and astrocytic pathophysiology. Using a 3-D in vitro model of cortical neurons from rats, Bar-Kochba et al. reported that a high-rate compressive dynamic load (SR = 75 s^−1^) causes a greater cytoskeletal deterioration and membrane blebbing than lower-rate loads (SR = 10 or 0.001 s^−1^)^43^. In addition, Cullen et al. used 3-D neuronal-astrocytic co-cultures of rats to investigate the effect of shear loads applied at different rates (i.e., SR = 1, 10, and 30 s^−1^) on cell viability and astrocytic alterations^61^. When compared to the lowest strain rate, they observed an increase in astrocytic hypertrophy and GFAP expression resulting from the higher strain rates. Moreover, their results indicated that the mode of astrocyte reactivity and the associated GFAP expression, as well as the extent of cell death, depend on the loading rate^61^. These observations support our finding that blast-induced changes in SR are strongly associated with molecular changes in the rat brain.

Multiples studies on rats have provided compelling evidence connecting changes in GFAP expression with neurobehavioral disorders resulting from blast exposure. Indeed, relative to controls, Kamnaksh et al. observed acute (at 2 h post-exposure) and chronic (at 22 days post-exposure) increases in GFAP expression in the hippocampus of rats exposed to a single 138 kPa BOP^35^. In addition, they observed that, at several time points ranging from 1 to 22 days post-exposure, the animals exhibited reduced mobility and impaired learning. In a separate longer-term study on rats, the same group reported similar molecular and neurobehavioral outcomes at 60 days post-exposure^62^. Separately, Sajja et al. assessed changes in GFAP and working memory in rats at 7, 30, and 90 days post-exposure to a single incident blast wave of 117 kPa^38,63^. They observed that, when compared to controls at 7 days post-exposure, exposed animals exhibited an approximate sixfold increase in GFAP in the prefrontal cortex, as well as compromised working memory^63^. Similarly, they reported as much as 3- and twofold increases in GFAP in the hippocampus and prefrontal cortex, respectively, at 30 days post-exposure and a concomitant disruption in learning and memory consolidation. Collectively, these results suggest that exposure to a blast wave triggers an astrocytic response, presumably to assist in the recovery of damaged cerebral tissue^53,64^, linked to the observed brain dysfunction. In addition, these findings highlight the relevance of GFAP as a potential biomarker of blast-induced brain injury and associated neurobehavioral disorders, which have been observed in military personnel with an occupational history of recurring exposure to blast waves^4–7,65,66^.

Our study has limitations. First, as acknowledged in our previous works^27,28,30,40^, we did not represent small-size vasculature (e.g., capillary vessels) in our computational models. However, provided that accurate 3-D geometries and material properties of these small brain vessels are available, we can potentially incorporate them into our computational models and more comprehensively account for the biomechanical responses at the interfaces of tissues with different capillary densities. Second, we determined the biomechanical correlates based on only one immunohistochemical assessment, GFAP staining. We selected GFAP because it allowed for the evaluation of brain cells (i.e., astrocytes) that are affected by blast exposure^8,49^. However, we acknowledge that analyses based on other proteins may yield different correlations. Third, our findings are based on GFAP changes observed at 24 h after blast exposure. Nonetheless, our methodology can be extended to scale mid- and long-term changes resulting from blast exposure. Fourth, the rat-to-human BOP scaling factors are specific to the head-on blast-exposure orientation considered in our study. As suggested by our previous study^30^, the orientation of the head with respect to the direction of the blast-wave propagation influences the biomechanical responses of the brain tissue. Therefore, we expect that the scaling factors would change when different head orientations are considered. Fifth, similar to other studies^28,29,67–69^, we investigated the biomechanical responses in the brain only for the first 5 ms of the blast-wave propagation and did not represent the negative-pressure phase. While we acknowledge that the deviatoric responses (e.g., strain) in the brain may evolve further after this initial period, the objective of our study was to characterize the responses in the brain resulting from the initial interaction of the blast wave with the head, which potentially leads to the so-called primary blast injury. Finally, due to the lack of immunohistochemical clinical data from humans, we were not able to verify the projected blast-induced changes in GFAP in the human brain. Alternatively, we would need to verify our methodology using an intermediate species, such as pigs or non-human primates.

To conclude, we combined experimental and computational studies and developed a new methodology to scale blast-induced molecular changes in the rat brain to the human brain. From our results, we determined that, compared to the rat, the human requires exposure to a blast wave of higher magnitude to elicit similar GFAP responses in the brain tissues, suggesting that the human brain is more resilient to biomechanical and molecular changes from blast insults. We also determined scaling factors from rats to humans. Hence, given a BOP and the corresponding GFAP level associated with an observed neurological impairment in rats, by multiplying the BOP by the scaling factor, we could identify the blast exposure in humans that yields the same “critical” BOP-induced change. Using such a methodology, we could develop dose–response curves and health-hazard guidelines that define thresholds for safe human exposure to blast waves.

## Supplementary Information

Supplementary Information.

## Data Availability

All data will be made available following a written request to the corresponding author, along with a summary of the planned research.
