# Supplementary material for: A biomechanical-based approach to scale blast-induced molecular changes in the brain
Source: Sci Rep. 2022 Aug 26;12:14605. doi: 10.1038/s41598-022-17967-6 (PMC9418170; doi:10.1038/s41598-022-17967-6)
Supplement: Supplementary file 1 — Supplementary Information. [file 41598_2022_17967_MOESM1_ESM.pdf]

A biomechanical-based approach to scale blast-induced molecular changes in the brain

Jose E. Rubio[1,2], Dhananjay Radhakrishnan Subramaniam[1,2], Ginu Unnikrishnan[1,2], Venkata Siva Sai Sujith Sajja[3], Stephen Van Albert[3], Franco Rossetti[3], Andrew Frock[1,2], Giang Nguyen[1,2], Aravind Sundaramurthy[1,2], Joseph B. Long[3], and Jaques Reifman[1*]

[1]Department of Defense Biotechnology High Performance Computing Software Applications Institute, Telemedicine and Advanced Technology Research Center, United States Army Medical Research and Development Command, Fort Detrick, MD 21702, USA

[2]The Henry M. Jackson Foundation for the Advancement of Military Medicine, Inc., 6720-A Rockledge Drive, Bethesda, MD 20817, USA

[3]Blast Induced Neurotrauma Branch, Center for Military Psychiatry and Neurosciences, Walter Reed Army Institute of Research, 503 Robert Grant Ave, Silver Spring, MD 20910, USA

Number of supplementary tables: 2

Number of supplementary figures: 3

Supplementary Material

Supplementary Table S1. Summary of incident blast overpressure parameters (mean ± one standard deviation) measured in the shock-tube experiments

Dataset	Parameter		
	**Peak overpressure (kPa)**	**Impulse (kPa·ms)**	**Duration (ms)**
80 kPa (n = 5)	80.61 ± 5.11	152.33 ± 27.67	4.36 ± 0.23
100 kPa (n = 4)	94.91 ± 2.32	213.99 ± 19.00	5.05 ± 0.56
130 kPa (n = 5)	127.70 ± 19.49	220.18 ± 55.08	4.76 ± 0.28

Supplementary Table S2. Correlations computed using either the peak incident blast overpressure (BOP) or its impulse

**Biomechanical response**	**Adj. R^2 BOP metric**	
	**Peak pressure**	**Impulse**
Corpus callosum		
SR	0.66	0.20
ICP	0.58	0.63
VMS	0.56	0.20
MPS	0.53	0.21
Hippocampus		
SR	0.72	0.20
ICP	0.55	0.65
VMS	0.65	0.10
MPS	0.60	0.11
Brainstem		
SR	0.47	0.24
ICP	0.46	0.20
VMS	0.47	0.46
MPS	0.38	0.31

Adj. R^2: adjusted R-squared; BOP: blast overpressure; ICP: intracranial pressure; MPS: maximum principal strain; SR: strain rate; VMS: von Mises stress.

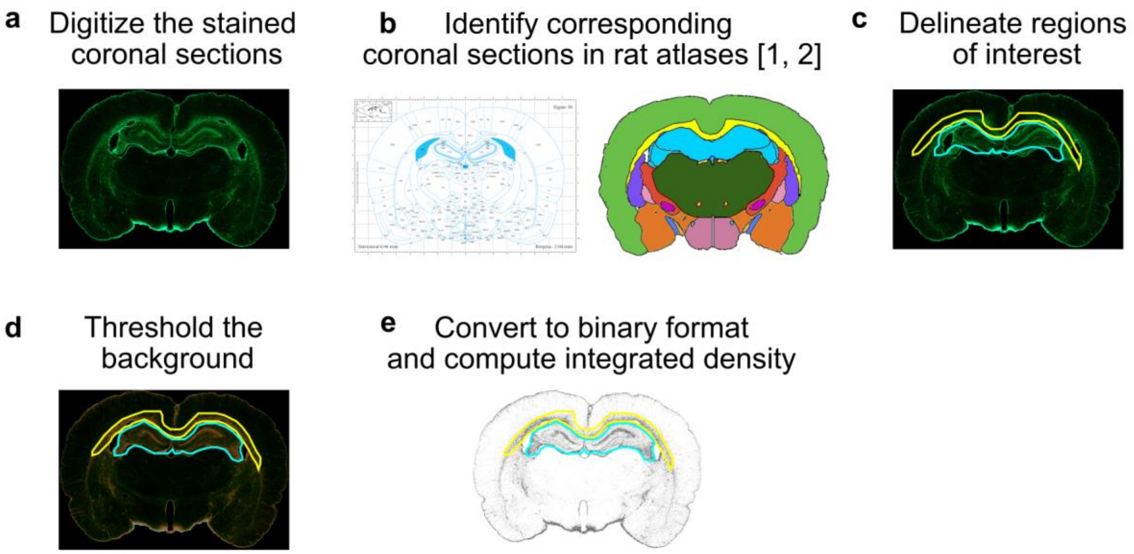

Supplementary Figure S1. Calculation of fluorescence intensity of GFAP-positive stained cells in different brain regions. (**a**) We digitized (10× magnification) each stained coronal brain section harvested from control and blast-exposed rats. Here, as an example, we show a stained coronal section located at −2 mm relative to Bregma. (**b**) Using rat-brain atlases [1, 2], we identified representative images of the same coronal sections used in the immunohistochemical analyses. (**c**) Using the atlas images as guides and the ImageJ software (National Institutes of Health, Bethesda, MD), we delineated the different brain regions in the stained coronal sections. In this figure, we show the corpus callosum in yellow and the hippocampus in blue. Note that the brainstem is absent at −2 mm relative to Bregma. Using the ImageJ software, (**d**) we removed the background pixels and (**e**) converted the image into a binary format. Lastly, we quantified the total intensity (i.e., the integrated density) of GFAP expression in three brain regions: corpus callosum, hippocampus, and brainstem. We repeated this procedure for each stained coronal section.

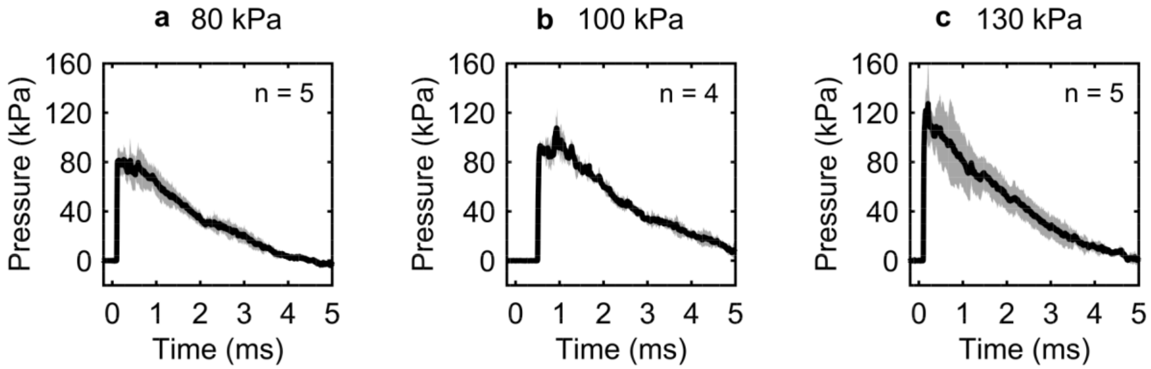

Supplementary Figure S2. Pressure-time profile of the incident blast overpressure (BOP) for the head-only blast-exposure experiments. We conducted head-only exposure on rats at targeted incident BOPs of (**a**) 80, (**b**) 100, and (**c**) 130 kPa. The solid lines and shaded areas represent the mean and one standard deviation, respectively.

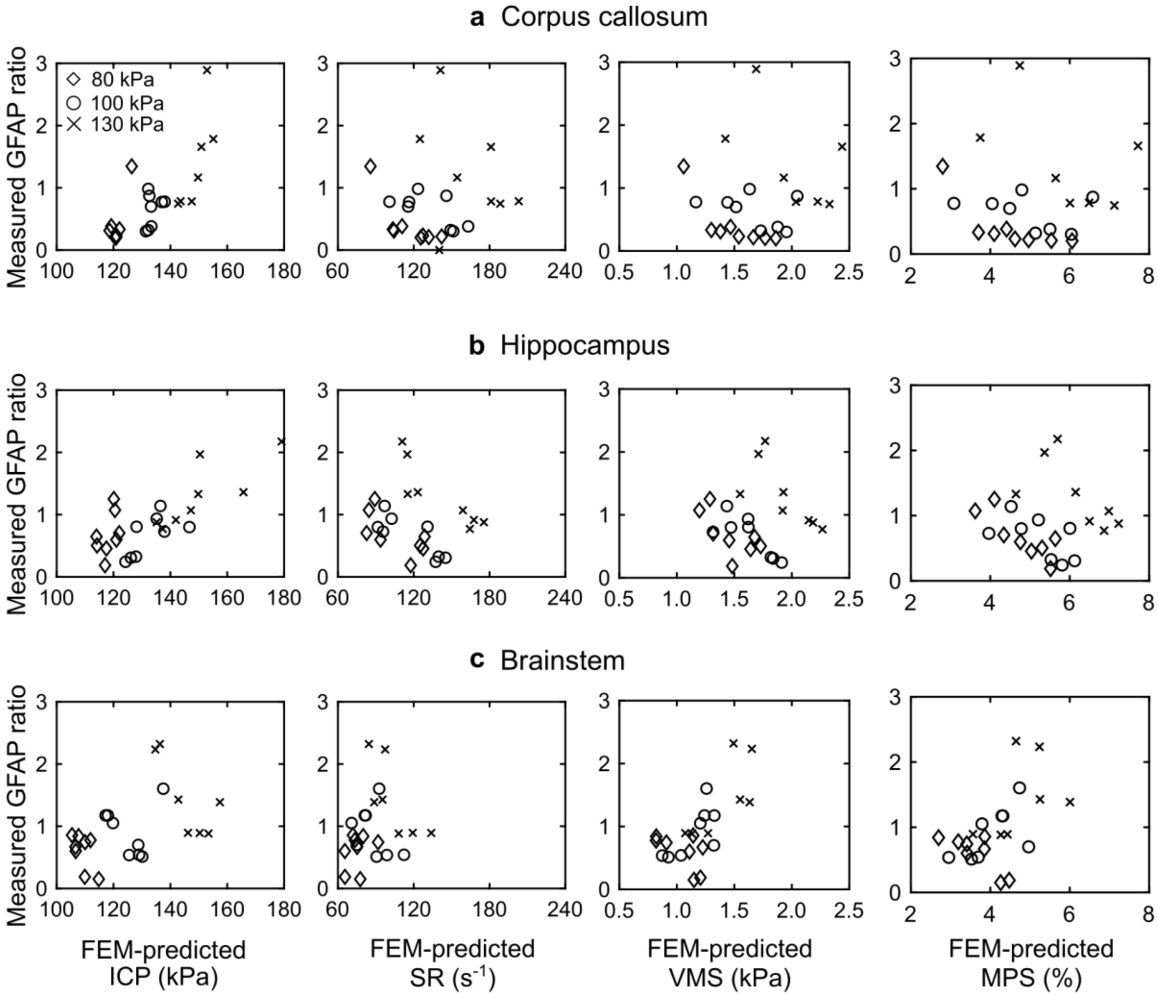

Supplementary Figure S3. Relationship between the experimentally measured GFAP changes and the computationally predicted biomechanical responses in the (**a**) corpus callosum, (**b**) hippocampus, and (**c**) brainstem resulting from a single head-only blast-wave exposure of rats at three blast overpressures (80, 100, and 130 kPa). Using the immunohistochemical data for each brain region within a coronal section, we computed a GFAP ratio by dividing the corresponding mean value of the head-only-exposed rats by that of the controls. Using the blast-simulation data for each brain region within a coronal section, we determined the peak 90th percentile of each biomechanical response over the entire simulation time (i.e., 5 ms). FEM: finite-element model; GFAP: glial fibrillary acidic protein; ICP: intracranial pressure; MPS: maximum principal strain; SR: strain rate; VMS: von Mises stress.

References

1. Paxinos, G. & Watson, C. *The Rat Brain in Stereotaxic Coordinates*. (Elsevier Science, 2013).
2. Papp, E. A., Leergaard, T. B., Calabrese, E., Johnson, G. A. & Bjaalie, J. G. Waxholm space atlas of the Sprague Dawley rat brain. *NeuroImage* **97**, 374-386, doi:10.1016/j.neuroimage.2014.04.001 (2014).
